# Help-Seeking Behavior for Children with Acute Respiratory Infection in Ethiopia: Results from 2011 Ethiopia Demographic and Health Survey

**DOI:** 10.1371/journal.pone.0142553

**Published:** 2015-11-11

**Authors:** Tigist Astale, Michelene Chenault

**Affiliations:** 1 Department of Health Education and Behavioral Sciences, Jimma University, Jimma, Ethiopia; 2 Department of Methodology & Statistics, Maastricht University, Maastricht, Netherlands; University of Tennessee Health Science Center, UNITED STATES

## Abstract

**Background:**

Acute respiratory infection is a major contributor to morbidity and mortality among children under five years of age in Ethiopia. While facilities have been implemented to address this problem they are underused due to a lack in help-seeking behavior. This study investigates factors related to the help-seeking behavior of mothers for children with acute respiratory infection using data from the 2011 Ethiopia Demographic and Health Survey.

**Methods:**

Data on 11,030 children aged 0–59 months obtained through interviewing women aged 15–49 years throughout Ethiopia was available. Descriptive statistics and logistic regression analyses were performed to determine which factors are related to help-seeking behavior for acute respiratory infection.

**Results:**

In the two weeks prior to the survey, 773(7%) of the children were reported to have symptoms of acute respiratory infection while treatment was sought for only 209 (27.2%). The odds ratio for acute respiratory infection was 1.6 (95% CI: 1.2–2.0) for rural residence with only 25.2% of these mothers seeking help compared to 46.4% for mothers with an urban residence. Smaller family size, younger mothers’ age and having had prenatal care had a statistically significant odds ratio greater than 1 for both urban and rural residences. Highest wealth index had a statistically significant odds ratio greater than 1 for rural residence only, whereas primary education or higher had a statistically significant odds ratio greater than 1 for urban residence.

**Conclusions:**

Children from rural areas are more at risk for acute respiratory infection while their mothers are less likely to seek help. Nevertheless, there is also underuse of available services in urban areas. Interventions should target mothers with less education and wealth and older mothers. Expanding prenatal care among these groups would encourage a better use of available facilities and subsequently better care for their children.

## Introduction

Although child mortality has been declining worldwide as a result of child survival interventions and socio-economic development [1], there were still 7.6 million children who died in the first 5 years of their life in 2010 [2]. Of the 7.6 million child deaths that occurred worldwide in 2010, pneumonia, diarrhea, and malaria were the leading causes of death. The largest burden of mortality in children younger than five years was reported in Africa (3.6 million) and Southeast Asia (2.1 million).

Acute respiratory infections (ARIs) are the most frequent childhood illnesses in low-income and middle-income countries [3,4]. ARIs can occur in any part of the respiratory system, from the middle ear to the nose and to the lungs. Pneumonia is a severe form of ARI that specifically affects the lungs [5]. Of all deaths in children younger than 5 years old, 1.4 million deaths were the result of pneumonia, accounting for 18.3% of total deaths in 2008. Ethiopia has an estimated 3.9 million cases of child pneumonia, with 112,000 dying, each year [4], making pneumonia the leading cause of death among small children. Pneumonia accounts for 28% of the yearly deaths, followed by newborn conditions (25%), diarrheal diseases (20%), and malaria (20%) [6].

Three essential steps are required for effective treatment of pneumonia among children under 5 years of age: recognizing that the child is sick, seeking appropriate care and a timely provision of a full course of antibiotic treatment [5]. Caretakers play a critical role in recognizing the symptoms of pneumonia and promptly seeking appropriate care. However, only 1 of every 5 caretakers in developing world is aware of the two foremost symptoms of pneumonia: fast breathing and difficult breathing [5]. Care seeking behavior for pneumonia is lower in Sub-Saharan Africa (41%), than in the Middle East and North Africa (66%) and East Asia and Pacific, excluding China, (62%) [5]. Particularly in Ethiopia, treatment from a health facility was sought for only 27% of children under five years of age with ARI symptoms [7].

To improve care for pneumonia, the Federal Ministry of Health of Ethiopia has introduced a community-based pneumonia treatment by health extension workers facilitating an upscaling of integrated Community Case Management of common childhood illnesses (iCCM) throughout the country. Community case management of pneumonia, complementing facility-based case management, is a strategy to deliver antibiotics outside of health facilities where there is limited access to treatment [8]. While this strategy has led to a significant improvement in the use of health care, this service remains underutilized [7,9]. The main reasons which have been reported for mothers not using iCCM services were: the mother thought that the child would get better (42%), domiciliary responsibilities of the mother (25%), financial reasons (16%), and not accustomed to seeking care (10%) [9].

It has been proposed that poor socio-economic status is associated with inadequate utilization of primary health care services [10–12]. Moreover, age and educational status of mothers appear to be related to health care seeking behavior. Mothers younger than 35 years and also mothers who have completed secondary education exhibit more help seeking behavior for their sick children [10,13]. At the same time, early marriage, which is common in Ethiopia [7], results in girls bearing children at an early age without having acquired sufficient maturity to care for their children’s health. The use of professional antenatal care has also been reported as a behavioral input to child health, in that, when mothers use routine professional antenatal care their children tend to have healthy outcomes throughout infancy and childhood [14]. The sex and age of the child have been shown to be associated with mothers’ help seeking behavior. It has been reported that care seeking is common for sick children in the youngest age group (0–11 months) and is slightly higher for boys than girls [10].

Factors promoting ‘good’ health care seeking behavior are not rooted solely in the individual but also have dynamic, collective and interactive aspects. Environmental factors such as the presence of health care facilities staffed adequately with health professionals and treatment possibilities, the availability of transport, physical distance to the facility, and time needed to reach the facility substantially influence health care seeking behavior [15–17].

In developing countries including Ethiopia, the effect of distance on service use is exasperated when distance is combined with lack of transportation and poor roads, increasing the burden of health care facility visits [15–20]. Moreover, location affects the availability of health facilities. Children residing in rural places have poorer access to vaccination and basic health care services which, if available, avert the highest toll of death from easily preventable and treatable diseases. For instance, vaccination coverage for children under 5 years of age has been reported at 48% in urban areas while only 20% in rural areas [7].

There are just a few studies on the help-seeking behavior of mothers for their children in Ethiopia. Given that, in Ethiopia, ARI among small children is a grave problem, the current study aims to assess help-seeking behavior of mothers for children with ARI and factors related to it. The goal is to assist policy makers and decision makers in designing interventions to promote help-seeking behavior for children with symptoms of ARI.

## Methods and Materials

This study utilized data from the third and most recent 2011 Ethiopia Demographic and Health Survey, nationally representative cross sectional household survey designed to obtain demographic and health indicators. The data set is publicly available online for all researchers and was obtained by contacting MEASURE DHS (Measure Demographic and Health Survey) program office. For this study survey data on 11,030 children aged 0–59 months, collected by interviewing women aged 15–49 years throughout Ethiopia, was available. The cases where childhood acute respiratory infection was reported (history of cough and difficulty in chest-related breathing in the two weeks prior to the interview) were considered for further analysis.

Help-seeking behavior was defined by the mother having contacted health services (hospital, health center, health station/clinic and health post excluding pharmacy, drug store, shop and traditional healer). The independent factors/variables considered were: age and sex of child, residence (rural/urban), and mother’s age, educational status and socioeconomic status according to a wealth index, family size and number of children under 5 years in the household. For the present study the five wealth quintiles (lowest, second, middle, fourth, and highest) were further recoded into three categories; lowest (by merging lowest and second categories), middle, and highest (by merging fourth and highest categories).

To operationalize environmental factors related to help-seeking behavior of caretakers, the mothers were asked whether the distance to the nearest health facility and having to take transport is a “big problem”, “not a big problem”, and “not a problem at all”. Distance to health facility and having to take transport were reduced in to two categories as “not a big problem” and “big problem”.

### Data analysis

The outcome variable was whether or not the mother had sought help for their child with ARI symptoms. Logistic regression analyses were performed to obtain crude ORs and 95% confidence intervals for each of the independent variables. Also, the logistic regression model was estimated when all of the independent variables considered were included simultaneously to obtain adjusted ORs (AORs) and 95% confidence intervals to check for potential cofounding, when determined analysis was re-done separately according to the confounding factor. SPSS software version 20.0 was employed.

### Ethics Statement

We utilized a publicly available Demographic and Health Survey data set by contacting MEASURE DHS program office. Demographic and Health Surveys follow standardized data collection procedures. The goals of the survey were explained to the respondents during data collection and written informed consent was obtained from mothers/caretakers on behalf of the children enrolled in the survey.

## Results

A total of 11,030 children were included in the survey. Of the 11, 030 children studied, 773 (7%) were reported to have symptoms of ARI in the two weeks prior to the interview, of these 773 children only 209 (27.2%) were taken to a health facility for care. Of these 773 children with ARI, 703 (91%) of them were from rural areas indicating that the proportion of ARI is disproportionately higher in rural areas, with the OR for ARI being 1.56 (95% CI: 1.21–2.01). Background characteristics are presented in Table 1. In Table 2, crude ORs and AORs are presented. The following were found to have statistically significant ORs greater than 1: OR = 1.44 (95% CI:1.04,2.01) for having just one child under 5 years in the household versus more than one child under 5, OR = 1.88 (95% CI:1.36,2.59) for small family size which is 2 to 5 persons versus 6 or more; OR = 2.66 (95% CI:1.91,3.71) for high wealth index versus middle and low; OR = 2.44 (95% CI:1.50,3.97) for being a young mother aged 15–24 years versus 35 years or more and OR = 2.00 (95% CI: 1.29–3.08) for mother’s age being between 25 and 34 years versus 35 years or more, and OR = 2.75 (95% CI: 1.89,3.98) for having received prenatal care. However, an urban residence was no longer statistically significant after adjustment for the other independent variables. Therefore the relation between all the independent variables and residence being either rural or urban were investigated with chi-square tests. In Table 3, frequencies relative to residence and the results of the chi-square analysis are presented. There was a highly significant relation between residence and number of children under 5 years, family size, wealth index, mother’s education, having had prenatal care, distance to a health facility and transport being a problem. It was therefore decided to perform the logistic regression analysis separately for urban and rural residences.

**Table 1:** Counts of help-seeking behavior for children with acute respiratory infection in Ethiopia (N = 773).

Characteristics	Help sought for ARI^1^	
	No	Yes
**Child sex**		
Male	293	100
Female	265	109
**Child age in months**		
0–11	118	37
12–23	86	34
24+	304	117
**# children < age 5 years**		
1	173	82
2	286	95
3+	93	31
**Family size**		
2–5	217	114
6+	340	95
**Residence**		
Urban	37	32
Rural	522	176
**Wealth index**		
Lowest	269	66
Middle	148	43
Highest	141	99
**Mothers’ education**		
No formal education	411	136
Primary education+	147	73
**Mothers’ age**		
15–24 years	122	61
25–34 years	278	115
35+ years	159	33
**ANC** ^2^ **from a skilled provider** ^3^		
No	319	99
Yes	95	82
**Distance to a health facility**		
Big problem	457	158
Not a big problem	101	51
**Having to take transport**		
Big problem	483	172
Not a big problem	75	37

^1^acute respiratory infection;

^2^antenatal care;

^3^skilled provided indicates doctor, nurse or midwife

**Table 2:** Odds ratios and 95% confidence interval for help-seeking behavior for children with acute respiratory infection in Ethiopia (N = 773).

Characteristics	COR^1^(95%CI)	AOR^2^(95%CI)
**Child sex**		
Male	Ref.	Ref.
Female	1.21(0.88–1.67)	1.10(1.00–3.66)
**Child age in months**		
0–11	Ref.	Ref.
12–23	1.26(0.73–2.16)	1.42(0.68–2.45)
24+	1.22(0.80–1.86)	0.93(0.59–1.61)
**# children < age 5 years**		
1	Ref.	
2	0.70(0.49–1.00)	
3+	0.71(0.44–1.15)	
**Only child below 5 years**	1.44(1.04,2.01)*	1.08 (0.70,1.65)
**Family size**		
2–5	1.88(1.36–2.59)*	1.18(0.76–4.81)
6+	Ref.	Ref.
**Residence**		
Urban	2.61 (1.58–4.31)*	1.13 (0.55–2.36)
Rural	Ref.	Ref.
**Wealth index**		
Lowest	Ref.	Ref.
Middle	1.19(0.77–1.83)	1.30(0.77–2.18)
Highest	2.84(1.96–4.12)*	3.70(2.17–6.27)*
**Wealth index high**	2.66(1.91, 3.71)*	2.76 (1.73,4.40)*
**Mothers’ education**		
No formal education	0.67(0.55–1.16)	1.48(0.93–2.34)
Primary education+	Ref.	Ref.
**Mothers’ age**		
15–24 years	2.44(1.50–3.97)*	1.92(1.01–3.66)*
25–34 years	2.00(1.29–3.08)*	2.49(1.44–4.31)*
35+ years	Ref.	Ref.
**ANC** ^3^ **from a skilled provider** ^4^		
No	Ref.	Ref.
Yes	2.75(1.89–3.98)*	2.16(1.40–3.33)*
**Distance to a health facility**		
Big problem	Ref.	Ref.
Not a big problem	1.47(1.01–2.16)*	1.43(0.75–2.73)
**Having to take transport**		
Big problem	Ref.	1.56(0.75–3.25)
Not a big problem	1.40(0.91–2.15)	Ref.

*significant finding for *P* values <0.05;

^1^Crude odds ratio;

^2^Adjusted odds ratio for child sex, child age, only child below five years, family size, high wealth index, mothers’ education, mothers’ age, antenatal care from a skilled provider, distance to health facility, and having to take transport;

^3^antenatal care;

^4^skilled provided indicates doctor, nurse or midwife

**Table 3:** Counts (percentages) of factors investigated relative to urban/rural residence.

Background characteristic		Urban (n = 69)	Rural (n = 703)	Chi-sq(df)	p-value
**Child age in months**	0–11	14 (21.9)	141 (22.1)	3.482 (2)	0.175
	12–23	6 (9.4)	117 (18.3)		
	24+	44 (68.8)	380 (59.6)		
**Child sex**	Male	37 (52.9)	356 (50.6)	0.125 (1)	0.723
	female	33 (47.1)	347(49.4)		
**# children < age 5 years**	1	32 (47.1)	222 (31.9)	7.081(2)	0.029
	2	25 (36.8)	361 (51.9)		
	3+	11 (16.2)	113 (16.2)		
**Only child under 5 years**				6.232	0.013
**Family size**	2–5	33 (47.8)	299 (42.5)	17.344	< 0.0005
	6–9	23 (33.3)	359 (51.1)		
	10+	13 (18.8)	45 (6.4)		
**Wealth index**	Low	6 (8.7)	330 (46.9)	116.288 (2)	< 0.0005
	Middle	2 (2.9)	194 (27.6)		
	High	61 (88.4)	179 (25.5)		
**Mother education**	No formal education	36 (52.2)	575 (73.2)	13.511 (1)	< 0.0005
	Primary or more	33 (47.8)	189 (26.8)		
**Age mother**	15–24	16 (22.9)	169 (24.0)	0.900 (2)	0.638
	25–34	33 (47.1)	360 (51.2)		
	35+	21 30.0)	175 (24.9)		
**ANC from skilled provider**	No	17 (33.3)	404 (73.9)	36.765	< 0.0005
	Yes	34 (67.7)	143 (26.1)		
**Distance to health care facility a big problem?**	Yes	44 (63.8)	576 (81.9)	13.114	< 0.0005
	No	25 (36.2)	127 (18.1)		
**Transport a problem?**	Yes	44 (63.8)	616 (87.6)	13.114	< 0.0005
	No	25 (36.2)	87 (12.4)		

For both urban and rural residences, smaller family size and having had prenatal care were statistically significant contributors to help-seeking for ARI (Table 4). For urban residences mother’s age less than 25 years was a predictor of help-seeking while for rural areas this was mother’s age less than 35 years. For urban residence, mothers who had at least had primary education were also more likely to seek help. There was no indication of confounding when considering the urban and rural sample separately.

**Table 4:** Odds ratios and 95% confidence intervals for help seeking behavior separately for urban and rural residence.

	Urban		Rural	
	OR	95% CI	OR	95% CI
**Child sex = female**	1.05	0.41–2.71	1.25	0.88–1.76
**Child age (ref: 0–11 mo)**				
12–23	1.38	0.19–9.82	1.35	0.76–2.41
24+	0.77	0.23–2.60	1.28	0.81–2.04
**#children < age 5 (ref: 3+)**				
1	4.36	0.91–20.99	1.19	0.71–1.98
2	3.15	0.63–15.74	0.89	0.55–1.46
**Only child under age 5 yrs**	1.92	0.74–5.00	1.31	0.91–1.87
**Family size < 6**	8.39*	2.85–24.69	1.57*	1.11–2.21
**Wealth index (ref: low)**				
Middle	28.54	0.24–76.02	1.14	0.73–1.77
High	2.50	0.36–17.13	2.63*	1.76–3.95
**Wealth Index High**	1.07	0.25–4.64	2.51*	1.74–3.63
**Mothers’ education (ref: none)**				
Primary education+	2.86*	1.08–7.59	1.26	0.86–1.83
**Mothers’ age (ref: 35+)**				
15–24 years	17.80*	2.81–112.71	2.18*	1.28–3.72
25–34 years	1.21	0.38–3.88	2.21*	1.37–3.55
**ANC from a skilled provider**	15.64*	2.86–85.67	2.22*	1.48–3.32
**Distance to a health facility not a big problem**	0.74	0.27–1.98	1.51	0.99–2.30
**Having to take transport not a big problem**	1.16	0.44–3.10	1.21	0.73–1.99

*significant finding for *P* values <0.05

## Discussion

Help-seeking behavior of mothers for children with symptoms of ARI is low in Ethiopia (27%) when compared to reported rates in developing countries (54%) and in Sub-Saharan Africa (41%) [5]. Here we found that help- seeking behavior of mothers was higher for mothers with the highest wealth index, conforming to findings from other studies in Kenya, Ethiopia, Nigeria, India, Ecuador and Bangladesh [10,12,21–24]. Ours study found that wealth index was a significant contributor to help- seeking by rural residents but not by urban residents. This might be explained by the indirect costs associated with distance from health facilities and having to take transport being more a concern for rural than urban residents. Making health facilities work for the poor, that is providing means to access, is mandatory to attain Millennium Development Goals [25]. Clearly attention must be paid to the costs the individual must incur to use available health care facilities in rural settings.

In our study we found that mother’s age influences help- seeking behavior. Help-seeking is higher for younger mothers (15–34 years) than for older mothers (35+ years) for rural residents whereas for urban areas mothers younger than 25 years exhibited more help-seeking. It has been reported by another study that younger women are more likely to be exposed to mass media than older women primarily because their level of education is higher [7], which might have contributed to a better help seeking behavior among younger mothers. The fact the mother’s age group (25–34 years) did not have a statistically significant OR in the urban setting may be due to the smaller urban sample. In any case age is associated with help-seeking behavior.

Smaller family size was also a statistically significant contributor to help- seeking for ARI for both rural and urban residences. Having a smaller family size possibly allows parents to invest more time and money in their sick child. The effect of smaller family size on help-seeking was reflected in both urban and rural residences, but this effect was larger for urban residences. This may be because average family size for urban household is 3.7 persons, compared to 4.9 persons in rural areas. Help- seeking was also higher for mothers who had attended antenatal care from a skilled provider for both rural and urban residences. This finding is in line with a study conducted in Nepal which suggested that when mothers use routine professional antenatal care their children tend to have healthy outcomes through infancy and childhood [14] which may be attributed to maternal health education.

Other factors for help-seeking behavior such as beliefs, predilections, ability to recognize symptoms of ARI and cultural factors were not investigated but should be considered when applying a more comprehensive conceptual model of health care utilization for a complete understanding of help seeking of mothers for suspected ARI.

### Methodological Strengths and Limitations

The findings can be generalized at the country level since the study utilized data from a nationally representative household survey. However, children were classified as having ARI only based on the experience of signs and symptoms of ARI reported by mothers without validation by medical. The other limitation is that some of the articles used for comparison used a period of signs and symptoms for ARI different from two weeks prior to the survey.

An additional limitation to be considered is the fact that in a survey such as this, respondents are asked about past events. Therefore the potential effect of recall bias on our results cannot be ignored.

## Conclusions

Help-seeking behavior of mothers for children with acute respiratory infection is low in Ethiopia. Interventions should target mothers with less education and wealth and older mothers. Expanding prenatal care among these groups would encourage a better use of available facilities and thus better care for their children.
